# Sleep Deficiency Is Associated With Exacerbation of Symptoms and Impairment of Anorectal and Autonomic Functions in Patients With Functional Constipation

**DOI:** 10.3389/fnins.2022.912442

**Published:** 2022-07-07

**Authors:** Jie Liu, Wei Wang, Jiashuang Tian, Chaolan Lv, Yuhan Fu, Ronnie Fass, Gengqing Song, Yue Yu

**Affiliations:** ^1^Department of Gastroenterology, Affiliated Anhui Provincial Hospital, Anhui Medical University, Hefei, China; ^2^Division of Life Sciences and Medicine, Department of Gastroenterology, The First Affiliated Hospital of University of Science and Technology of China (USTC), University of Science and Technology of China, Hefei, China; ^3^Division of Gastroenterology and Hepatology, MetroHealth Medical Center, Case Western Reserve University, Cleveland, OH, United States

**Keywords:** constipation, autonomic dysfunction, anorectal function, anxiety, depression, sleep deficiency

## Abstract

**Objective:**

Sleep deficiency (SD) is commonly seen in patients with functional constipation (FC). Our aim was to determine whether the presence of SD would influence symptoms, anorectal motility, sensation, and autonomic function in FC patients.

**Materials and Methods:**

A total of 85 FC patients with SD and 193 FC patients without SD underwent high-resolution anorectal manometry. SD was assessed by using the Pittsburgh Sleep Quality Index (PSQI) score. Participants were required to fill in the entire questionnaires, including Patients’ Constipation-symptoms, State-Trait Anxiety Inventory, and Hamilton Depression Scale. Autonomic dysfunction was studied by recording the heart rate variability. Multiple logistic regression was performed to explore the potential risk factors for anorectal function.

**Results:**

Functional constipation patients with SD had a higher total score of constipation symptom (*P* < 0.001), in comparison with those without SD. FC patients with SD demonstrated significantly lower threshold volume for first sensation (*P* < 0.001) and urge (*P* < 0.001), as compared to those without SD. The PSQI score positively correlated with constipation symptom total score (*P* < 0.001), and negatively correlated with threshold volume for first sensation (*P* < 0.001) and urge (*P* < 0.001). FC patients with SD had a reduced vagal activity (*P* = 0.016) and a higher sympathetic activity as compared to those without SD (*P* = 0.003). Multivariate logistic regression revealed that SD, anxiety and depression were independent risk factors for anorectal function, with SD exhibiting the highest degree of association with first sensation (OR: 4.235).

**Conclusion:**

Sleep deficiency is associated with worse constipation related symptoms, altered anorectal function and perception, and impaired autonomic function in FC patients.

## Introduction

Sleep deficiency (SD) is defined as subjects not getting enough sleep and/or sleeping at an inappropriate time of day (Hyun et al., 2019; Orr et al., 2020). SD is frequently associated with disorders of gut-brain interaction (DGBI), such as irritable bowel syndrome (IBS), functional dyspepsia, and chronic constipation (Orr et al., 2020). SD is related to various gastrointestinal symptoms, including abdominal pain, acid reflux, abdominal distension, and belching (Hyun et al., 2019). Previous studies have shown that lack of sleep is associated with an increased risk of multiple gastrointestinal complaints and decreased quality of life (Jiang et al., 2017; Orr et al., 2020). For example, DGBI is associated with excessive daytime sleepiness in a study involving 3600 Chinese patients (Wu et al., 2017). Moreover, SD is directly related to gastrointestinal dysfunction and symptoms (Chen et al., 2011; Schurman et al., 2012). IBS patients with SD are found to have anorectal dysfunction (Chen et al., 2011).

Although SD has been associated with DGBI, the relationship of SD with anorectal function remains unclear due to conflicting data in the literature. Several studies have indicated that SD affects gastrointestinal motility in patients with DGBI. Park et al. (2020) reported lower melatonin concentrations in sleep-deprived mice compared to the control group. Furthermore, the metagenomic analysis of microbiota indicated an abundance of colitogenic microbiota in sleep-deprived mice. Therefore, the authors concluded that intestinal dysbiosis could be influenced by sleep deprivation, resulting in increased colitogenic microbiota, which could aggravate colonic dysfunction. Haase et al. (2015) found that basal colonic activity was suppressed during both deep sleep and light sleep compared to nocturnal wake periods *via* 3D-Transit system. Besides, suppressed basal colonic activity was detected during both deep sleep (*P* < 0.05) and light sleep (*P* < 0.05) when compared with nocturnal wake periods. However, Liu et al. (2010, 2011) showed that SD did not affect anorectal function in healthy subjects.

Sleep deprivation is closely related to autonomic dysfunction (Tobaldini et al., 2017). It has been shown that autonomic dysfunction is closely associated with the development of functional constipation and enhancement of parasympathetic activity could significantly improve symptoms of functional constipation (FC) (Chen et al., 2018, 2021). Furthermore, pro-inflammatory cytokines are released in the setting of acute sleep deprivation, which might result in recurrent symptoms in patients with inflammatory bowel disease and IBS (Axelsson et al., 2013). SD is also linked to anorectal dysfunction and creates some degree of rectal hyperalgesia in patients with IBS (Chen et al., 2011). The interaction between SD and altered anorectal function may be multi-factorial and needs further investigation.

Recent studies showed that patients with FC had significantly lower sleep quality compared with patients with IBS (Chen et al., 2020). However, the relationship between SD and its impact on anorectal function remains largely unknown in patients with FC. The aim of this study was to investigate the impact of patient-reported SD on symptoms, anorectal function, and rectal sensitivity in patients with FC.

## Materials and Methods

### Patients

Patients who met the Rome IV diagnostic criteria (Drossman, 2016) for FC were recruited into the study at the Gastrointestinal Motility Center, Department of Gastroenterology, the First Affiliated Hospital of University of Science and Technology of China (USTC), from November 2016 to January 2020. The exclusion criteria were: severe cardiac and pulmonary diseases, diabetes, chronic kidney disease, and other chronic gastrointestinal disorders such as inflammatory bowel disease, peptic ulcer disease, and cancer. Meanwhile, patients reporting a chief complaint of abdominal pain were ruled out given that abdominal pain inherently distinguishes FC with IBS-C (Ruffle et al., 2021). Our study excluded patients taking pain modulators and patients with severe mental diseases, such as patients with depression scores higher than 24, suggesting severe depression. In particular, patients who were taking chronic opioids and non-sleep aid medications that could potentially affect sleep quality were excluded. These non-sleep aid medications include the following: (i) antiasthmatic medications, such as aminophylline, doxofylline, and ephedrine; (ii) antidepressants, such as paroxetine, fluoxetine, and imipramine; (iii) antibiotics, such as penicillin, macrolides, quinolones, and (iv) glucocorticoid.

**FIGURE 1:**
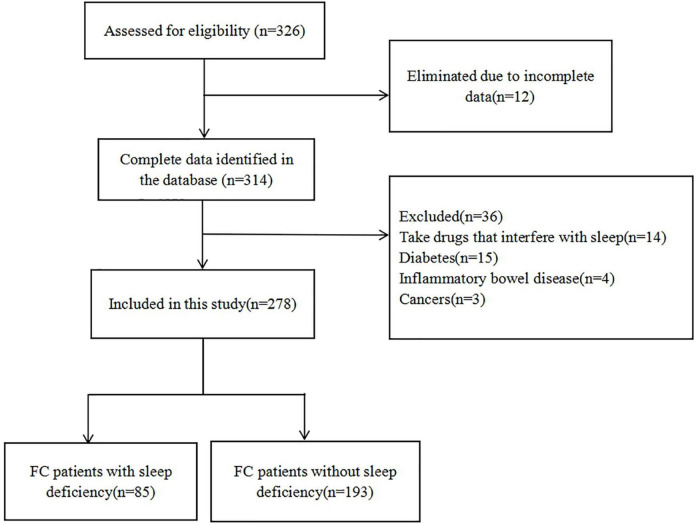
Flow chart of FC patient recruitment in this study.

A total of 326 FC patients were eligible for the study. Twelve patients were excluded from the study due to incomplete data. Thirty-six patients were also excluded based on the exclusion criteria (14 patients who took medications that affect sleep quality, 15 patients with diabetes, 4 patients with IBD, and 3 patients with colorectal cancer). Finally, a total of 278 FC patients (114 males and 164 females) were included in this study (Figure 1).

The study was approved by the Ethics Committee of Anhui Provincial Hospital (Registration No: 2022-RE-143). The study protocol was registered in the Chinese Clinical Trial Registry (No. ChiCTR-2000037449). Written informed consent was obtained from all participants before their enrollment into the study.

### Experimental Protocol

This is a cross-sectional cohort study, and patients with FC were divided into two groups based on the Pittsburgh Sleep Quality Index (PSQI) scores: FC patients with SD (*n* = 85) (PSQI scores ≥ 5) and FC patients without SD (*n* = 193) (PSQI scores < 5).

All participants were required to fill in the entire questionnaires, including PSQI, Patients’ Constipation-symptoms (PAC-SYM), State-Trait Anxiety Inventory (STAI), and Hamilton Depression Scale (HAMD). All FC patients underwent high resolution anorectal manometry (HRAM) and received electrocardiogram (ECG) recording for heart rate variability analysis.

### Measurements

#### High Resolution Anorectal Manometry

All FC patients underwent HRAM (MedKinetic, Ningbo, China), and the process of HRAM was described in our previous research (Liu et al., 2020). Each patient received 1–2 doses of glycerin enema for bowel preparation 30–60 min prior to the HRAM test. Rectal enema is the preoperative preparation for anorectal manometry, in order to avoid the residue of stool in anorectal, which affects the acquisition of dynamic parameters. A water-perfused anorectal manometric catheter was used to measure the anal sphincter pressure at a 1-cm interval. The device employs the technology of proprietary pressure transduction, which allows every pressure-sensor element to sense pressure over a 2.5-mm length in each of the twelve sectors that are dispersed radially. Patients were placed in the left lateral position and the catheter was inserted into the rectum after lubrication. Manometry parameters, including average resting pressures, maximum and sustained squeeze pressures, were analyzed by the manometry software (Manometryapp, MedKinetic, Ningbo, China). The pressure of the anal sphincter was examined during voluntary effort, while the study participants were asked to squeeze the anus for as long as possible to record squeeze pressures.

The threshold volume for rectoanal inhibitory reflexes (RAIR) was examined through inflation of the balloon in a stepwise manner to 10 ml, beginning at 10 ml until we observed relation of the anal sphincter at a lower distension volume. The sensation test was examined by the rectal balloon distended at a 10 ml interval until the participant indicated the first sensation. After that, we increased the volume of the balloon in progressive 10-ml increments so that the participant felt the sensations of the urge and the maximum urge to defecate. The results for inducing these sensations were depended on the subjects’ report subjectively, and the threshold volumes for this sensory test were documented. Rectal compliance of every subject was calculated from the slope of the volume-pressure curve, which was automatically analyzed by the manometry software (Manometryapp, MedKinetic, Ningbo, China) (Lee and Bharucha, 2016).

#### Questionnaires

The PSQI as a tool for diagnosing SD was employed to assess the quality of sleep in the past month. The PSQI is split into seven dimensions, namely, subjective sleep quality, daytime dysfunction, sleep latency, use of sleep medication, sleep duration, SD, and habitual sleep efficiency. Each dimension is scored on a four-point Likert scale (0–3, from none to the most profound effect). A total score of 5 or more suggests “SD.” The PSQI has shown a specificity of 86.5 and 89.6% sensitivity using this cutoff point (Chen et al., 2011; Liu et al., 2011).

The PAC-SYM questionnaire was used in the psychometric assessment of patients with chronic constipation. This questionnaire evaluated the severity of symptoms in patients with FC. The survey included 12 items divided into 3 symptom subscales, including rectal (3 items), stool (5 items), and abdominal (3 items). The rectal domain collected information about pain, burning, tearing, or bleeding during bowel movements. The abdominal domain evaluated discomfort, bloating, pain, and stomach cramps. The stool domain was categorized as hard, small, incomplete, straining, and difficult bowel movement. The items of this scale were scored through five-point Likert scales, ranging from 0 to 4. In this case, 0 represented the absence of symptoms, while 4 indicated very severe symptoms. A low average score showed a low symptom burden (Frank et al., 1999).

The STAI was adopted for assessing the levels of anxiety (Guillén-Riquelme and Buela-Casal, 2014). STAI is a 20-item instrument scored on a four-point Likert-type scale (1–4, from not at all to very much). Healthy subjects without anxiety have total scores < 40 (Vigneau and Cormier, 2008). HAMD was adopted for assessing the levels of depression. HAMD is a 17-item instrument and uses a 5-point Likert scale (0–4, from no symptom to severe symptoms). A global HAMA score of >7 indicates “depression” (Lu et al., 2020).

#### Assessment of Autonomic Functions

The functions of the autonomic nervous system of the subjects were measured with spectral analysis of HRV (heart rate variability), as described in our previous research. The HRV analysis software V.1.2.0.0 (Cardiotrak Holter system; Hangzhou Baihui Electrocardiograms, China) was employed to analyze each subject’s HRV data, whereas HRV signals were provided by using an electrocardiogram (ECG) recording (ct-082, Hangzhou Baihui Electrocardiograms, China). Calculation of the power in every frequency sub-band was employed to determine the power spectral analysis. In general, parasympathetic or vagal activities are represented by the band of high frequency (0.15–0.50 Hz, HF), while the power in the band of low frequency (0.04–0.15 Hz, LF) primarily represents sympathetic activity. Furtherly, the HF/(HF + LF) ratio was used to represent parasympathetic activity. Meanwhile, the Baevsky Index or Sympathetic Index (SI) was calculated to represent sympathetic tone according to the formula $SI=\frac{AMo\times 100\%}{2Mo\times \mathrm{MxDMn}}$. The most frequent RR interval was transformed into the mode (Mo), which is expressed in seconds (Ali et al., 2021). A 50 ms bin width was used for calculating the amplitude of mode (AMo), which is expressed as a percentage of the total number of intervals measured. MxDMn represented the variability as the difference between longest (Mx) and shortest (Mn) RR interval values, expressed in seconds. The SI is expressed as s^–2^.

#### Statistical Analysis

All the statistical analyses were implemented in the SPSS V.16.0 software. Continuous variables are given as mean ± standard deviation. Statistical comparisons were investigated using normality testing, followed by paired *t*-test. Pearson’s correlation coefficient were used to assess the relationship among PSQI, anorectal function and constipation symptoms. Moreover, a multivariate logistic regression analysis was employed, including anorectal function as the dependent variables and all those variables with statistically significant differences in the bivariate analysis as independent variables. Anxiety and depression were considered as potential confounding factors. *P* < 0.05 signified statistical significance.

## Results

### Overall Study Population

Finally, a total of 278 FC patients who underwent HRAM were enrolled for this study, including 85 FC patients with SD (accounting for 30.58% in FC) and 193 FC patients without SD. All patients tolerated the procedures without any adverse effects. No remarkable differences were reported between the groups regarding age, BMI, as well as, gender and there was no difference in duration of constipation between the FC subgroups (*t* = 1.548, *P* = 0.123) (Table 1).

**TABLE 1:** Characteristics of the FC population (*N* = 278).

Variables	FC patients with SD (*n* = 85)	FC patients without SD (*n* = 193)	χ^2^/*t*	*P*
**Gender**				
Male (*n*)	38	76	0.692	0.405
Female (*n*)	47	117		
Age (years; mean ± SE)	47.39 ± 9.58	46.64 ± 10.09	0.576	0.565
BMI (kg/m^2^; mean ± SE)	24.34 ± 4.29	23.61 ± 3.91	1.397	0.164
Duration of constipation (months; mean ± SE)	34.92 ± 7.71	33.25 ± 8.42	1.548	0.123

*Data are expressed as mean ± standard deviation.*

*FC, functional constipation; SD, sleep deficiency; BMI, body mass index.*

*No statistically significant difference was noted in age, gender, BMI among the two groups.*

### Constipation Symptom and Anxiety/Depression Score

Functional constipation patients with SD had a higher PAC-SYM score than group FC patients without SD (15.720 ± 1.493 vs. 12.750 ± 1.339, *P* < 0.001) (Table 2). Specifically, FC patients with SD had a higher abdominal symptom score (4.670 ± 1.084 vs. 4.340 ± 0.876, *P* = 0.008) and higher rectal symptom score (4.890 ± 0.926 vs. 4.560 ± 0.882, *P* = 0.005) compared with FC patients without SD. However, there was no difference in defecation symptoms score between the two groups (4.550 ± 0.838 vs. 4.410 ± 0.886, *P* = 0.207). Meanwhile, there was also no significant difference in the anxiety and depression scores between FC patients with SD and those without SD (*P* = 0.411 and *P* = 0.451, respectively) (Table 2).

**TABLE 2:** Constipation symptom, anxiety, and depression in FC patients with or without SD.

	FC with SD	FC without SD	*t*	*P*
**PAC-SYM**				
Abdominal symptoms	4.67 ± 1.08	4.34 ± 0.88	2.673	0.008
Rectal symptoms	4.89 ± 0.93	4.56 ± 0.88	2.824	0.005
Defecation symptoms	4.55 ± 0.84	4.41 ± 0.89	1.266	0.207
Total score	15.72 ± 1.49	12.75 ± 1.34	16.424	<0.001
STAI total score	37.18 ± 9.16	36.17 ± 9.48	0.823	0.411
HAMD total score	6.18 ± 2.08	5.99 ± 1.92	0.754	0.451

*Data are expressed as mean ± standard deviation.*

*FC, functional constipation; SD, sleep deficiency; PAC-SYM, patient assessment of constipation symptoms; STAI, State-Trait Anxiety Inventory; HAMD, Hamilton Depression Scale.*

### Anorectal Function

When compared to FC patients without SD, FC patients with SD had a significantly lower threshold volume for the first sensation (22.00 ± 3.87 vs. 24.48 ± 3.85, *P* < 0.001), the urge to defecate (106.71 ± 9.92 vs. 114.97 ± 9.08, *P* < 0.001), and maximal defecation (123.18 ± 10.69 vs. 141.63 ± 11.50, *P* < 0.001). Conversely, FC patients with SD had a significantly higher threshold volume for the RAIR (17.46 ± 4.15 vs. 16.12 ± 4.84, *P* = 0.019). FC patients with SD had lower anal sphincter pressure for maximal squeeze than that of FC patients without SD (143.14 ± 14.12 vs. 151.03 ± 11.87, *P* < 0.001). However, there was no difference in length of anal sphincter and compliance between FC patients with SD and those without SD (*P* = 0.800 and *P* = 0.685, respectively) (Table 3).

**TABLE 3:** Anorectal function in FC patients with or without SD.

	FC with SD	FC without SD	*t*	*P*
**Threshold volume, ml**				
First sensation	22.00 ± 3.87	24.48 ± 3.85	4.94	<0.001
Urge	106.71 ± 9.92	114.97 ± 9.08	6.80	<0.001
Maximal	123.18 ± 10.69	141.63 ± 11.50	12.59	<0.001
RAIR	17.46 ± 4.15	16.12 ± 4.84	2.36	0.019
**Anal sphincter pressure, mm Hg**				
Resting	56.06 ± 5.52	55.67 ± 5.47	0.54	0.590
Maximal	143.14 ± 14.12	151.03 ± 11.87	4.81	<0.001
Sustained squeeze	197.34 ± 7.99	201.53 ± 7.82	0.083	0.934
Length of anal sphincter, cm	2.25 ± 0.29	2.23 ± 0.33	0.254	0.800
Compliance, ml/mm Hg	6.18 ± 0.67	6.15 ± 0.63	0.406	0.685

*Data are expressed as mean ± standard deviation.*

*CF, functional constipation; SD, sleep deficiency; RAIR, rectoanal inhibitory reflexes.*

### Multivariate Logistic Regression Analysis

Results of the logistic regression are shown in Table 4. In summary, In summary, among all the potential factors included in the model, SD (OR: 4.235) and anxiety (OR: 1.743) showed a statistically significant influence on the first sensation, while SD (OR: 2.496) showed an influence on the urge to defecate. In addition, SD (OR: 3.147) and depression (OR: 3.024) showed a significant influence on maximal defecation, while SD (OR: 2.145) showed a statistically significant influence on RAIR.

**TABLE 4:** Multivariate logistic regression analysis about the potential factors of anorectal function for FC patients.

Potential factors	First sensation			Urge			Maximal			RAIR		
	OR	95%CI	*P*	OR	95%CI	*P*	OR	95%CI	*P*	OR	95%CI	*P*
Age	0.953	0.691–1.453	0.162	0.867	0.603–1.215	0.174	0.783	0.596–1.269	0.246	0.961	0.711–1.608	0.144
Gender	1.023	0.768–1.957	0.092	1.105	0.794–2.013	0.084	0.983	0.631–1.857	0.272	1.314	0.823–1.965	0.063
SD	4.235	2.018–7.869	**0.001**	2.496	1.836–3.871	**0.018**	3.147	1.981–4.746	**0.005**	2.145	1.768–2.939	**0.041**
Anxiety	1.743	1.106–2.459	**0.015**	0.768	0.987–1.416	0.496	1.006	0.536–1.896	0.355	0.793	1.018–2.153	0.403
Depression	1.412	0.926–2.728	0.052	1.245	0.871–2.669	0.073	3.024	1.168–6.739	**0.003**	1.306	1.002–2.781	0.064

*OR, odds ratio; CI, confidence interval; RAIR, rectoanal inhibitory reflexes; FC, functional constipation; SD, sleep deficiency. The bold values indicate P < 0.050.*

### Correlations Between Pittsburgh Sleep Quality Index and Other Parameters

**FIGURE 2:**
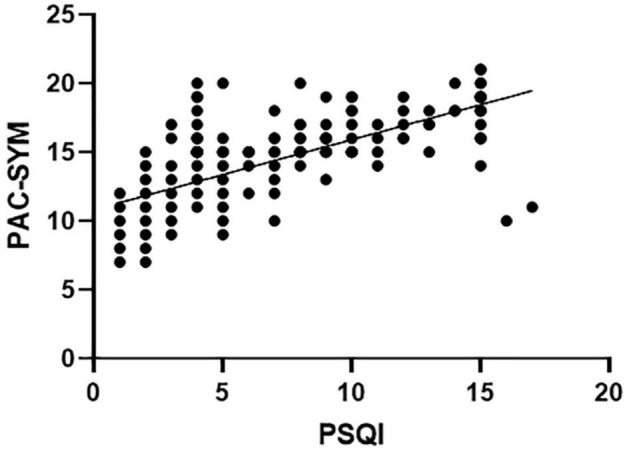
Sleep quality scores (PSQI) significantly correlated with constipation symptom scores (PAC-SYM) in FC patients (Pearson Coefficient = 0.686, *P* < 0.001).

Pittsburgh sleep quality index significantly correlated with total score of PAC-SYM (*r* = 0.686, *P* < 0.001), as well as abdominal and rectal subscores (*r* = 0.194; *P* = 0.001 and *r* = 0.156; *P* = 0.009, respectively), but there was no correlation between PSQI and defecation symptoms (*r* = –0.041, *P* = 0.501). PSQI negatively correlated with the threshold volume for the first sensation (*r* = –0.330; *P* < 0.001), urge to defecate (*r* = –0.366; *P* < 0.001), maximal defecation (*r* = –0.671; *P* < 0.001), and RAIR (*r* = 0.323; *P* < 0.001) in patients with FC. PSQI also negatively correlated with maximal squeeze (*r* = –0.233; *P* = 0.001) despite the lack of correlation between PSQI and resting and sustained squeeze in FC patients (*r* = 0.054; *P* = 0.366 and *r* = –0.074; *P* = 0.219, respectively) (Table 5). The scatter plot between PSQI and PAC-SYM score was shown in Figure 2.

**TABLE 5:** Correlations between PSQI and constipation symptom as well as anorectal function in FC patients.

	FC	
	*r*	*P*
**Threshold volume, ml**		
First sensation	–0.330	<0.001
Urge	–0.366	<0.001
Maximal	–0.671	<0.001
RAIR	0.323	<0.001
**Anal sphincter pressure, mm Hg**		
Resting	0.054	0.366
Maximal	–0.233	0.001
Sustained squeeze	–0.074	0.219
Length of anal sphincter, cm	0.048	0.422
Compliance, ml/mm Hg	0.040	0.509
**PAC-SYM**		
Abdominal symptoms	0.194	0.001
Rectal symptoms	0.156	0.009
Defecation symptoms	–0.041	0.501
Total score	0.686	<0.001

*Data are expressed as Pearson’s correlation coefficient with p values in parentheses.*

*PSQI, Pittsburgh Sleep Quality Index; FC, functional constipation; RAIR, rectoanal inhibitory reflexes; PAC-SYM, patient assessment of constipation symptoms.*

### Mechanisms Involving Autonomic Functions

**FIGURE 3:**
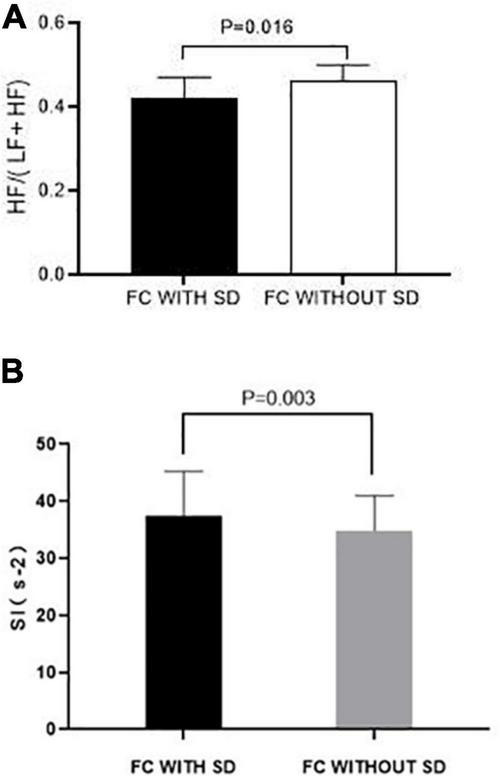
Effects of SD on autonomic function in FC patients. **(A)** Parasympathetic activity represented as the ratio of HF/(LF + HF) in FC patients with SD was significantly lower than that of FC patients without SD (*P* < 0.050). **(B)** Sympathetic activity represented as the Baevsky Index or Sympathetic Index (SI) in FC patients with SD was significantly higher than that of FC patients with SD (*P* < 0.050).

Functional constipation patients with SD had a significantly lower parasympathetic activity when compared to that of FC patients without SD [HF/(HF + LF) ratio, 0.42 ± 0.05 vs. 0.46 ± 0.04, *P* = 0.016] (Figure 3A). Meanwhile, FC patients with SD had a higher sympathetic activity than that of FC patients without SD (SI, 37.48 ± 7.75 vs. 34.88 ± 6.09, *P* = 0.003) (Figure 3B).

## Discussion

In this study, we investigated the influence of SD on symptoms, anorectal motility, sensation in FC patients by a validated sleep questionnaire (PSQI) and HRAM, and explored potential mechanisms using spectral analysis of HRV. We found that FC patients with SD had more severe constipation symptoms (especially abdominal and rectal symptoms) than those without SD. Meanwhile, FC patients with SD had a significantly lower sensory threshold for anorectal balloon distension and a lower anal sphincter pressure for the maximal squeeze. Notably, we also showed that SD increased sympathetic activity while simultaneously suppressed parasympathetic activity. Results from multivariate logistic regression analysis shown that SD was significant independent risk factors for anorectal function with first sensation exhibiting the highest degree of association, while anxiety was independent risk factors for first sensation and depression was independent risk factors for maximal defecation.

Although the relationship between SD and DGBI is incompletely understood, latest studies indicated that there were close associations of SD with lower gastrointestinal symptoms. In a cross-sectional internet-based survey for Japanese population, Yamamoto et al. (2021) tested the relationship between chronic constipation and sleep and the authors demonstrated that subjects with constipation and poor sleep experienced severe symptoms and had poor quality of life, which is consistent with our findings. Jiang et al. (2017) investigated 126 patients with chronic constipation and reported that patients with sleep disorders had worse constipation symptoms evaluated by the constipation scoring system (CSS) scale, compared to those without sleep disorders (*P* = 0.031). Latest study evaluated the sleep quality in patients with FC and constipation-predominant IBS (IBS-C), and the results showed that FC patients had worse sleeping quality than IBS-C patients (Chen et al., 2020). In this study, we found that FC patients with SD patients were characterized by more severe abdominal and rectal symptoms than those without SD. However, no remarkable differences were reported between the groups regarding defecation symptoms, which may be because included patients already had severe defecation alterations for a long-term course based on Rome IV criteria. Meanwhile, our study showed that the PSQI significantly correlated with the abdominal (*P* = 0.001) and rectal symptoms (*P* = 0.009) in FC patients with SD.

While the influence of sleep dysfunction on anorectal function and motility was investigated in previous studies for IBS patients, the impact of SD on anorectal functions and motility in FC has been scarcely studied. To our best knowledge, it is the first study to determine whether the presence of SD would influence symptoms, anorectal motility, sensation, and autonomic function in patients with FC. By application of HRAM, we observed that FC patients with SD had a lower threshold volume for the first sensation, maximal defecation, urge to defecate, and a significant increase in the threshold volume for RAIR when compared to FC patients without SD, which is similar to previous findings (Chen et al., 2011; González Cañete et al., 2014). FC patients with SD had a lower threshold volume for the first sensation, maximal defecation, urge to defecate when compared to FC patients without SD. What is more, FC patients with SD had a significant decrease in the maximal squeeze pressure, which may due to autonomic dysfunction (Iino et al., 2004). Meanwhile, our study showed that the PSQI significantly correlated with the perception threshold to anorectal balloon distension in FC patients with SD.

The PSQI serves as a major tool to evaluate sleep quality during the past month. Previous studies have shown that the PSQI score correlates with some mental health diagnoses, including anxiety, stress, depression, and psychotic disorders (Zhao et al., 2018). Anxiety as well as other psychological indicators might influence the relationship of the perception of SD with visceral hypersensitivity (Meerveld and Johnson, 2018). Thus, it might be controversial that if the poor quality of sleep combined with anxiety and/or other psychological comorbidities but not SD itself influences rectal sensitivity as noted in this research. However, no difference in STAI and HAMD scores was observed between FC patients with and without SD. Besides, multivariate logistic regression analysis was applied, to elucidate the association among anorectal function, SD, anxiety and depression. Results showed that SD, anxiety and depression were significant independent risk factors for anorectal function, with SD exhibiting the highest degree of association with first sensation (OR: 4.235). Therefore, it is more likely that poor sleep quality most significantly affects anorectal function in FC patients, although other comorbidities, consisting of stress and negative events, might influence rectal sensitivity (Parker et al., 2019).

It is known that autonomic dysfunction plays an important role in the pathogenesis of FC (Mazur et al., 2012). In the current study, HRV analysis was used to represent the autonomic cardiac function, which can be explored as a substitute for autonomic nerve function. Furtherly, this approach has been previously employed to demonstrate that a sympathetic to parasympathetic post-prandial ratio decreases with an increase in vagal activity (Bruinstroop et al., 2013). What is more, alterations in cardiac autonomic functions triggered by meal opine it can be a surrogate for gastrointestinal autonomic function (Wang X. et al., 2019). It has been reported that poor sleep will lead to sympathetic activation. Wang S. et al. (2019) reported that poor sleep would increase the LF/HF ratio and make LF higher, reflecting increased sympathetic activity and decreased parasympathetic activity. Carter et al. (2018) reported that individuals with chronic insomnia exhibit increased sympathetic neural as well as cardiovascular reactivity to stress, augmented sympathetic neural outflow, and blunted baroreflex sensitivity relative to good-sleeper individuals. Of note, Liu et al. (2018) documented that an escalation in sympathetic nerve activity is associated with constipation. Meanwhile, our previous study showed that the imbalance between sympathetic and parasympathetic nerves plays a vital role in constipation symptoms (Liu et al., 2020). Notably, the current study showed that sleep disorders will influence the balance between parasympathetic and sympathetic nerves thus remarkably increasing sympathetic activities, which is similar outcomes to previous research (Carter et al., 2018; Wang S. et al., 2019).

On the other hand, visceral hypersensitivity has been reported by the incidence of functional gastrointestinal conditions, e.g., non-cardiac chest pain, IBS, as well as heartburn (Simrén et al., 2018). Visceral hypersensitivity is linked to insufficient sleep, while SD could promote visceral perception conversely. Moreover, the interrelation between visceral hypersensitivity and SD has been regarded as an etiological event of chronic hyperalgesia syndromes (Lee, 2006). Although previous study have shown that sleep disorders cannot result in anorectal motility changes in healthy people (Liu et al., 2011), Whitehead et al. (1990) found that patients with IBS have high chances of reporting the first rectal pain sensation at lower pressures compared to healthy individuals. To date, there is no report on the relationship between constipation and visceral hypersensitivity. In the current study, we found that FC patients were recognized by a lower sensory threshold to anorectal balloon distension. We believe that SD is associated with impairment of anorectal functions in FC patients and this change may be related to autonomic nerve function and visceral hypersensitivity. However, it is worth mentioning that the data regarding volume differences between the groups presented in our study were small statistical changes. This may suggest our patients with FC plus SD were likely associated with more severe anorectal hypersensitivity by their ability to differentiate a small balloon volume changes. SD may play a role in the pathogenesis of visceral hypersensitivity in patients with FC. Fass et al. (2000) demonstrated that the visceral sensitivity of functional bowel disorder patients can be aggravated by sleep maintenance disorders. Consequently, SD may be involved in the mechanism behind these changes. Visceral hypersensitivity is linked to insufficient sleep, whereas SD could promote visceral perception. Moreover, the interrelation between visceral hypersensitivity and SD has been considered an etiological event of chronic hyperalgesia syndromes (Lee, 2006). Notably, the gut microbiota is another potential mechanism that affects the rectal sensitivity in patients with FC. Previous studies reported a decrease in beneficial bacteria such as Lactobacillus, an increase in harmful bacteria, and a reduction in species richness in FC patients (Wang and Yao, 2021). Colonic functions could be modulated by gut microbiota *via* the metabolites of bacterial fermentation, which could trigger the release of gut hormones. Subsequently, colonic sensation, secretion, and motility could be impacted by these gut hormones (Ohkusa et al., 2019; Ding et al., 2021). Overall, the relationship between SD and altered anorectal function in FC may be multi-factorial, and the underlying mechanism needs further investigation.

A systematic review put forward a new model of communication between DGBI and sleep microbial metabolites, including the serotonergic system, the vagus nerve, and immune reactions (Sen et al., 2021). Non-rapid eye movement (NREM) sleep was decreased by serotonin depletion in the brain during the inactive phase and was increased during the active phase in rats (Nakamaru-Ogiso et al., 2012). This suggests that serotonin might play an important role in communicating gut microbiome and sleep regulation in the brain. Meanwhile, vagotomized mice did not display sleep deprivation-associated inflammation, which demonstrated the role of the vagus nerve in crosstalk between the gut microbiota and sleep (Zhang et al., 2021). Regional homogeneity (ReHo) and resting-state functional magnetic resonance imaging scans were performed by Feng et al. (2022) revealing the relationships among ReHo values in the left fusiform gyrus, the relative abundance of Lactobacilli, and depression scores in chronic insomnia patients. In addition, some bacterial genera related to the ReHo values of the right triangular inferior frontal gyrus were also found; and the relative abundance of genus Coprobacter was correlated with the ReHo values of the left angular gyrus. These findings revealed complex relationships between DGBI and chronic insomnia. Schoch et al. (2022) reported a link between sleep habits and gut microbiota. They found that daytime sleep is related to bacterial diversity, while nighttime sleep fragmentation and variability are linked with bacterial maturity and enterotype. In addition, they proposed a sleep-brain-gut link, suggesting that sleep neurophysiology is related to bacterial diversity and enterotype. Another study has demonstrated that indigenous spore-forming microbes from the gut microbiota produced metabolites that promoted host 5-HT biosynthesis in the gastrointestinal tract and impacted gut motility (Yano et al., 2015). It is well known that 5-HT regulated gut motility and alterations in 5-HT signaling might contribute to FC.

## Limitations

There were some potential limitations in the present study. Firstly, although patients with serious mental diseases were excluded, patients with mild depression were included. Sleep issues may be related to or exacerbated by mental diseases and impact our results. Secondly, the healthy control group can be used as the baseline control, improving the comparison of the parameters of the water perfusion manometry system, such as the sensory test of FC patients with/without SD. However, it is difficult and ethically challenging perform a manometric examination in healthy people. Thirdly, the sensory test’s value was determined subjectively by the patients’ report, which might be disturbed by the patient’s status at the time of examination. Besides, HRAM with water perfused was used in the current study, while most recent studies are performed using solid state catheters. Previous studies reported no differences at rest between the two types of catheters, but solid-state catheters offer greater sensitivity to rapid pressure changes compared to water perfusion (Liem et al., 2012; Rasijeff et al., 2017). Thus, using a water-perfused HRAM catheter may lead to different results. Finally, another limitation includes the fact that other objective measures of colonic function (such as transit studies, etc.) were not included in the assessment of FC patients, and the patients did not undergo a formal mental health assessment before participating in the study. Although patients with serious mental diseases were excluded, sleep issues may be related or exacerbated by the mental diseases which can in turn impact our results.

## Conclusion

In summary, the present study verifies the previous concept of the relationship of subjective disturbances of sleep with gastrointestinal symptoms. We established that individuals with FC with SD are remarkably sensitive to rectal distention discomfort, and they exhibit evidence of changed anorectal function and severe constipation symptoms. The potential mechanisms may be related to autonomic nervous function and visceral hypersensitivity. However, it remains to be investigated if the mechanism(s) of SD affecting constipation, such as the central nervous system’s dysfunctions, affects intestinal function.

## Data Availability Statement

This datasets presented in this study can be found in online repositories. The names of the repository/repositories and accession number(s) can be found in the article/supplementary material.

## Ethics Statement

The study was approved by the Ethics Committee of Anhui Provincial Hospital (Registration No: 2022-RE-143). The study protocol was registered in the Chinese Clinical Trial Registry (No. ChiCTR-2000037449). Written informed consent was obtained from all participants before their enrollment into the study. Written informed consent was obtained from the individual(s) for the publication of any potentially identifiable images or data included in this article.

## Author Contributions

YY and GS planned the study. JL, WW, JT, CL, and YY performed HRAM. JL and CL collected and interpreted the data. JL drafted the manuscript. YY, GS, YF, and RF revised the manuscript critically. All authors read and approved the final manuscript.

## Conflict of Interest

The authors declare that the research was conducted in the absence of any commercial or financial relationships that could be construed as a potential conflict of interest.

## Publisher’s Note

All claims expressed in this article are solely those of the authors and do not necessarily represent those of their affiliated organizations, or those of the publisher, the editors and the reviewers. Any product that may be evaluated in this article, or claim that may be made by its manufacturer, is not guaranteed or endorsed by the publisher.
